# Family planning use among women living with HIV: knowing HIV positive status helps - results from a national survey

**DOI:** 10.1186/s12978-015-0035-6

**Published:** 2015-05-10

**Authors:** Dereje Habte, Jane Namasasu

**Affiliations:** United Nations Development Programme (UNDP)/United Nations Volunteers (UNV), Lilongwe, Malawi; Malawi Ministry of Health, Lilongwe, Malawi

**Keywords:** Family planning, HIV, Malawi, Women

## Abstract

**Background:**

Women living with HIV continues to encounter unintended pregnancies with a concomitant risk of mother-to-child transmission of HIV infection. Preventing unintended pregnancy among HIV-infected women is one of the strategies in the prevention of new HIV infections among children. The aim of this analysis was to assess the practice of family planning (FP) among HIV-infected women and the influence of women’s awareness of HIV positive status in the practice of FP.

**Methods:**

The analysis was made in the Malawi Demographic and Health Survey (DHS) data among 489 non-pregnant, sexually active, fecund women living with HIV. Multiple logistic regression analysis was performed using SPSS software to identify the factors associated with FP use. Adjusted odds ratios (AOR) with 95 % confidence intervals were computed to assess the association of different factors with the practice of family planning.

**Result:**

Of the 489 confirmed HIV positive women, 184 (37.6 %) reported that they knew that they were HIV positive. The number of women who reported that they were currently using FP method(s) were 251 (51.2 %). The number of women who reported unmet need for FP method(s) were 107 (21.9 %). In the multiple logistic regression analysis, women’s knowledge of HIV positive status [AOR: 2.32(1.54, 3.50)], secondary and above education [AOR: 2.36(1.16, 4.78)], presence of 3–4 alive children [AOR: 2.60(1.08, 6.28)] and more than 4 alive children [AOR: 3.03(1.18, 7.82)] were significantly associated with current use of FP.

**Conclusion:**

Women’s knowledge of their HIV-positive status was found to be a significant predictor of their FP practice. Health managers and clinicians need to improve HIV counselling and testing coverage among women of child-bearing age and address the FP needs of HIV-infected women.

## Introduction

Globally, 3.2 million children were living with HIV in the year 2013 and 190,000 children died of AIDS-related illnesses. In low and middle income countries, the number of children newly infected with HIV annually was estimated to be 240,000. Only 24 % of children in need of treatment were able to access the service [1]. Over 90 % of the children acquire HIV infection via mother-to-child transmission (MTCT) during pregnancy, delivery or breast feeding periods [2]. Without intervention, the risk of MTCT is in the range of 20–45 % whereas the risk of MTCT goes as low as 2–5 % with appropriate interventions [2].

In Malawi, a nationwide survey in 2010 indicated a national HIV prevalence of 10.6 % among adults (15–49 years): women at 12.9 % and men at 8.1 % [3]. Hetero-sexual intercourse (88 %) and MTCT (10 %) were the major modes of HIV transmission in Malawi [4]. A cohort study conducted in urban Malawi demonstrated that the total fertility rate (TFR) among women enrolled in anti-retroviral therapy (ART) was similar to the TFR in the general urban population [5]. Another longitudinal study done in Malawi in a different setting found out that pregnancy rates in HIV-infected and HIV-uninfected women were similar [6]. A qualitative study result from Malawi also showed that couples living with HIV continued giving birth although they were aware of the associated risk [7].

A significant proportion of parents living with HIV continued to desire to have children despite the risk of MTCT [8–11]. The desire to reproduce, lack of information on MTCT and poor outcome of previous pregnancies were among the factors that prompted parents living with HIV to desire children [9, 10]. Several HIV-positive women encountered unintended pregnancies with a concomitant risk of MTCT [12–16]. FP lessens the risk of unintended pregnancies among women living with HIV and hence reducing the chance of MTCT. The global PMTCT strategy and the Malawi national plan for the elimination of MTCT (EMTCT) advocates a four-pronged strategy of elimination of new HIV infections among children and keeping their mothers alive. The four pillars of the strategy are primary prevention of HIV infection, preventing unintended pregnancy among HIV-infected women, preventing HIV transmission from HIV-infected women to their infants, and care for HIV-infected mothers and their children [2, 17].

Studies have demonstrated that HIV-infected women who know their status have a lower fertility desire and better use of contraceptives as compared to their HIV-negative counterparts [6, 18–20]. A study done in Malawi indicated that women with longer follow up time on ART were associated with increased chance of becoming pregnant [5]. The effectiveness of contraceptive use in preventing HIV-positive births has already been established [21]. Contraceptive use is one of the strategies for the elimination of MTCT of HIV infection in Malawi [17].

Malawi has been implementing the EMTCT program since 2011 through the introduction of the Option B+ approach. A 2013 report shows that 82 % of the estimated pregnant women in the year were counseled and tested for HIV. Of the estimated 63,000 HIV‐positive pregnant women in the country during the year, 73 % received ART (Option B+). Malawi managed to test and provide results to only 30 % of HIV-exposed infants within 2 months of birth [4]. There is a gap in terms of providing HIV testing to all expected pregnancies, ART delivery to all possible HIV-infected women eligible for Option B+ and HIV testing to exposed infants. Over one-fourth of the estimated HIV-positive pregnant women did not receive ART and remained at higher risk of delivering HIV-infected babies. Contraceptive use can contribute to the EMTCT effort by filling in the mentioned gaps and by tackling unintended pregnancies among HIV-infected women.

Previous studies compared contraceptive use between HIV-infected individuals who were aware of their HIV status versus HIV-negative counterparts. In contrast, this analysis compared HIV-infected women who were aware of their status versus HIV-infected women who were unaware of their status. This study is different in that it tried to assess whether or not women’s awareness of their HIV-positive status affects their FP practice. The analysis was performed using Malawi DHS data of non-pregnant, sexually active, fecund women living with HIV collected in 2010. The aim of the analysis was to assess the practice of FP among HIV-infected women and the influence of women’s awareness of HIV-positive status in the practice of FP.

## Materials and methods

### Study area

Malawi is divided into three regions namely the Northern, Central, and Southern Region. There are 28 districts in the country. The Malawi DHS was implemented by the National Statistical Office (NSO) with a nationally representative sample of more than 27,000 households. Individual interviews were made with all eligible women aged 15–49 in these households and all eligible men aged 15–54 in a subsample of one-third of the households. HIV testing was conducted among eligible women aged 15–49 and eligible men aged 15–54 in a selected subsample of one-third of the households. Data for this analysis were drawn from the Malawi DHS of 2010 which was designed to provide population and health indicator estimates at the national, regional, and district levels [3].

### Data collection

Standard household questionnaire, women’s questionnaire and men’s questionnaire were used. The questionnaires were adapted to reflect the population and health issues relevant to Malawi. In addition to English, the questionnaires were translated into two major languages: Chichewa and Tumbuka. The questionnaires were pre-tested and data collectors were trained on the questionnaires, interviewing techniques and field procedures. Data were collected by thirty-seven interview teams. One supervisor (team leader), one field editor, four female interviewers, two male interviewers, and one driver constituted a team. Staff members from NSO and ICF Macro coordinated and supervised fieldwork activities. Data collection took place over six-month period: June-November 2010 [3].

In 2010, the Malawi DHS recorded data relating to fertility levels, nuptiality, sexual activity, fertility preferences, awareness and use of family planning methods, breastfeeding practices, nutritional status of mothers and young children, early childhood mortality, maternal mortality, maternal and child health, malaria, awareness and behaviour regarding HIV/AIDS and other sexually transmitted infections, and HIV prevalence. This analysis is based on 489 non-pregnant, HIV confirmed fecund women who were sexually active in the 12 months preceding the survey [3].

### HIV testing procedure

Eligible women and men were asked to voluntarily provide five drops of blood for HIV testing. Participants were provided with information on the procedure, confidentiality of the data, and the fact that the test results would not be made available. After securing consent for the HIV testing, five blood spots from the finger prick were collected on a filter paper card to which a bar code label unique to the respondent was affixed. Information brochure on HIV/AIDS was provided to each household irrespective of their consent to give blood for HIV testing.

The details of the testing protocol and handling of blood samples are described in Malawi DHS report [3].

### Ethical issues

The study protocol and the procedures for the blood specimen collection and the testing for HIV was reviewed and approved by the Malawi Health Sciences Research Committee, the Institutional Review Board of ICF Macro, and the Centres for Disease Control and Prevention (CDC) in Atlanta. All consent procedures in DHS are verbal consent. All three ethics committees/IRBs mentioned above approved this consent procedure. The identities of the respondents remained anonymous and the signatures of the respondents were not collected. An alternate procedure of obtaining consent was used: the interviewer read the consent statement to the respondent and the respondent was free to consent or not to consent. The interviewer signed his or her signature confirming that he or she read the consent statement to the respondent, and records the respondent’s reply (“yes voluntarily consents” or “no does not consent”).

Consent to individual interviews and HIV testing of adults was made by the respondents themselves and the interviewer signed his/her own name testifying that he/she read the informed consent statement to the respondents. Minors eligible for participating in the survey are those age 15–17. Informed consent for individual interviews and blood collection from the minors was obtained from the parents or guardians of the minors and from the minors themselves. When a minor consented, the interviewer went on to obtain voluntary consent from the parent or guardian. Consent from parents/guardians and minors for the individual interviews and blood collection was recorded by the interviewer signing his or her own name testifying that he/she read the consent statement to the guardian/minor. When consent was given, the interviewer signed his/her own name testifying that the informed consent statement was read and the respondent’s consent accurately recorded (“yes voluntarily consents” or “no does not consent”). The protocol allowed for the merging of the HIV test results with the socio-demographic data collected in the individual questionnaires, provided that identifier information of an individual was destroyed before data linking takes place [3].

### Outcome measure

Women were asked whether they or their partners were using a method of FP at the time of the survey. Women who reported current use of either modern or traditional contraceptive methods were considered as current users of FP method.

### Exposure measures

The potential predictors of current FP practice were grouped into four categories: socio-demographic, access to FP information, reproductive and awareness of HIV positive status. The details of the potential predictors are as follows:-

- Socio-demographic: age, education, marital status, region, residence (urban/rural), currently working (yes/no), and wealth Index
- Access to information: radio, television, newspaper, visit by FP worker at home, and woman’s visit to a health facility
- Reproductive: number of births in the past five years, number of under-five year child, number of children ever born, and number of living children
- Knowledge of HIV positive status: all women included in this analysis are those with confirmed HIV positive result. About 37.6 % of the women reported that they knew their HIV positive status.

Women who indicated that they either wanted no more children (limiters) or wanted to wait for two or more years before having another child (spacers), but were not using contraception, were identified as having an unmet need for FP [3].

### Data analysis

This analysis was performed on data from a selected group of sexually-active women in their reproductive age with HIV positive test results in the DHS. All women fulfilling the criteria in the three regions were included. Percentages and means were used to describe the characteristics of study participants. Bivariate analysis (Chi-square test) was used to assess the effect of each independent variable on current practice of FP. Multivariable logistic regression analysis was used to explore the effect of the different exposure variables on the outcome variable. Multiple logistic regression analysis was performed to control for possible confounding factors. The logistic regression analysis was made in three steps: first model using socio-demographic variables; second model included socio-demographic, access to health information and reproductive variables; the final model included women’s awareness of HIV positive status in addition to all the variables in the second model. Multiple logistic regression analysis (enter method) was used. Odds ratios with 95 % confidence interval (95 % CI) were computed for the association between risk factors and FP practice. Statistical significance was considered at p-value less than 0.05. IBM SPSS Statistics for Windows, Version 20.0. Armonk, NY: IBM Corp was used in the analysis of the data.

Detailed information on the study area, study population, organization of the survey, sample design, questionnaires, data collection, data quality, data processing and ethical issue is published in the Malawi DHS 2010 report [3].

The lead author communicated with MEASURE DHS/ICF International and permission was granted to download and use the data for this project.

## Results

A total of 489-laboratory confirmed HIV-infected women who were sexually active in the one year preceding the survey were included in the analysis. Over four-fifth of the women were aged 25 years and above. Women with no formal education, primary education and secondary/post-secondary education constituted 14.9 %, 63.8 % and 21.3 % respectively. The majority were married/cohabiting (71.8 %), from southern region (64.0 %), rural residents (74.0 %) and currently working (67.3 %). Women in the richer and richest category were 22.3 % and 32.1 % respectively whereas the proportions of the poorest, poorer and middle categories were 13.1 %, 15.7 % and 16.8 %. In the bivariate analysis, none of the socio-demographic variables were significantly associated with current practice of FP (P ≥ 0.05) [Table 1].

**Table 1:** The association between socio-demographic characteristics and current practice of family planning method(s) among HIV-infected fecund women

Characteristics		Total number (Column %)	Current practice of any FP		P-value*
			Yes (Row %)	No (Row %)	
Age group in years					
	15–24	77 (15.7)	43 (55.8)	34 (44.2)	0.63
	25–34	220 (45.0)	109 (49.5)	111 (50.5)	
	35–49	192 (39.3)	99 (51.6)	93 (48.4)	
	Mean (SD): 32.1 (7.3)				
Education					
	No education	73 (14.9)	29 (39.7)	44 (60.3)	0.09
	Primary	312 (63.8)	165 (52.9)	147 (47.1)	
	Secondary & above	104 (21.3)	57 (54.8)	47 (45.2)	
Marital status					
	Married/Cohabiting	351 (71.8)	190 (54.1)	161 (45.9)	0.05
	Others	138 (28.2)	61 (44.2)	77 (55.8)	
Region					
	Northern	67 (13.7)	42 (62.7)	25 (37.3)	0.09
	Central	109 (22.3)	50 (45.9)	59 (54.1)	
	Southern	313 (64.0)	159 (50.8)	154 (49.2)	
Residence					
	Urban	127 (26.0)	60 (47.2)	67 (52.8)	0.30
	Rural	362 (74.0)	191 (52.8)	171 (47.2)	
Currently working					
	Yes	329 (67.3)	168 (51.1)	161 (48.9)	0.92
	No	160 (32.7)	83 (51.9)	77 (48.1)	
Wealth index					
	Poorest	64 (13.1)	35 (54.7)	29 (45.3)	0.42
	Poorer	77 (15.7)	38 (49.4)	39 (50.6)	
	Middle	82 (16.8)	35 (42.7)	47 (57.3)	
	Richer	109 (22.3)	61 (56.0)	48 (44.0)	
	Richest	157 (32.1)	82 (52.2)	75 (47.8)	

*Chi-Square test

The proportion of women who accessed FP information via radio, television and newspapers were 65 %, 14.1 % and 16.6 % respectively. A significant majority of the respondents (83.6 %) were not visited by a FP worker at home in the year preceding the survey whereas 76.5 % of them reported to have visited a health facility in the past one year. In the five years preceding the survey, 44.6 % of the women did not give any birth, 38.9 % had one birth and 16.6 % reported two or more births. Over one-third of the participants did not have under-five children. Women who reported to have never given birth constituted 6.5 % of all the women. About 9.4 % of all the women did not have any alive child. Of the 489-laboratory confirmed HIV-infected women, 184 (37.6 %) knew their HIV positive status of which 92 (50 %) reported that they were on ARV treatment. In the bivariate analysis, visiting a health facility in recent time, all reproductive characteristics and knowledge of HIV positive status were statistically significantly associated with current practice of FP (P < 0.05) [Table 2].

**Table 2:** The association between family planning information, reproductive history, knowledge of HIV positive status and current practice of family planning method(s) among HIV-infected fecund women

Characteristics		Total number (Column %)	Current practice of any FP		P-value*
			Yes (Row %)	No (Row %)	
FP information from radio					
	Yes	318 (65.0)	170 (53.5)	148 (46.5)	0.21
	No	171 (35.0)	81 (47.4)	90 (52.6)	
FP information from Television					
	Yes	69 (14.1)	35 (50.7)	34 (49.3)	1.00
	No	420 (85.9)	216(51.4)	204 (48.6)	
FP information from newspaper					
	Yes	81 (16.6)	44 (54.3)	37 (45.7)	0.62
	No	408 (83.4)	207 (50.7)	201 (49.3)	
Visited by FP worker past 12 months					
	Yes	80 (16.4)	45 (56.3)	35 (43.8)	0.39
	No	409 (83.6)	206 (50.4)	203 (49.6)	
Visited health facility past 12 months					
	Yes	374 (76.5)	207 (55.3)	167 (44.7)	0.00
	No	115 (23.5)	44 (38.3)	71 (61.7)	
Births in past 5 years					
	0	218 (44.6)	87 (39.9)	131 (60.1)	0.00
	1	190 (38.9)	113 (59.5)	77 (40.5)	
	2 & more	81 (16.6)	51 (63.0)	30 (37.0)	
Number of under- 5 children					
	0	185 (37.8)	75 (40.5)	110 (59.5)	0.00
	1	197 (40.3)	115 (58.4)	82 (41.6)	
	2 & more	107 (21.9)	61 (57.0)	46 (43.0)	
No of children ever born					
	0	32 (6.5)	5 (15.6)	27 (84.4)	0.00
	1–2	135 (27.6)	69 (51.1)	66 (48.9)	
	3–4	164 (33.5)	85 (51.8)	79 (48.2)	
	5 & more	158 (32.3)	92 (58.2)	66 (41.8)	
No of living children					
	0	46 (9.4)	9 (19.6)	37 (80.4)	0.00
	1–2	193 (39.5)	103 (53.4)	90 (46.6)	
	3–4	158 (32.3)	85 (53.8)	73 (46.2)	
	5 & more	92 (18.8)	54 (58.7)	38 (41.3)	
Knows HIV positive status					
	Yes	184 (37.6)	119 (64.7)	65 (35.3)	0.00
	No	305 (62.4)	132 (43.3)	173 (56.7)	

*Chi-Square test

All women, with the exception of one, reported to have knowledge about modern contraceptives. Over four-fifth of them had ever used a modern contraceptive method where as 16.4 % never used any contraceptive method. About 0.6 % of the women reported to have ever used folkloric methods while 2.5 % said that they had ever used traditional methods. A total of 238 women (48.7 %) were not using any FP method at the time of the survey while 1.8 %, 2.0 % and 47.4 % were using folkloric method, traditional method and modern method respectively. Injectable contraceptive, condom and female sterilization were the mostly practiced current FP methods. Over one-fifth of the women were found to have unmet need for FP: 7.4 % unmet need for spacing children and 14.5 % unmet need for limiting pregnancy [Table 3].

**Table 3:** Knowledge and practice of family planning among HIV-infected fecund women

Characteristics		Frequency	Percentage
Knows modern contraceptives (n = 489)			
	Yes	488	99.8
	No	1	0.2
Ever used method class (n = 489)			
	Never used	80	16.4
	Folkloric	3	0.6
	Traditional method	12	2.5
	Modern method	394	80.6
Currently used method class (n = 489)			
	No method	238	48.7
	Folkloric method	9	1.8
	Traditional method	10	2.0
	Modern method	232	47.4
Current used method type (n = 489)			
	Not using	238	48.7
	Pill	4	0.8
	Injectable	96	19.6
	Condom	62	12.7
	Female Sterilization	54	11.0
	Male Sterilization	1	0.2
	Periodic Abstinence	4	0.8
	Withdrawal	6	1.2
	Norplant	13	2.7
	Female condom	2	0.4
	Other	9	1.8
Reasons for FP use (n = 489)			
	Using to space	83	17.0
	Using to limit	168	34.4
	Non-users	238	48.7
Reasons for not using FP (n = 489)			
	Using FP	251	51.4
	Unmet need to space	36	7.4
	Unmet need to limit	71	14.5
	Desire birth in less than 2 years	63	12.9
	No sex, want to wait	63	12.9
	No response	5	1.0

Bivariate analyses were performed to determine the association between socio-demographic variables and women’s knowledge of their HIV positive status. Education, marital status, region, residence, working status, wealth index and births in the past five years were not statistically significantly associated with women’s knowledge of their HIV positive status (p > 0.05). Older women were more likely to know their HIV positive status than younger women (p < 0.05) [Table 4].

**Table 4:** The association between socio-demographic characteristics and knowledge of HIV positive status among HIV-infected fecund women

Characteristics		Total number	Knows HIV positive status		P-value*
			Yes (Row %)	No (Row %)	
Age group in years					
	15–24	77	13 (16.9)	64 (83.1)	0.00
	25–34	220	86 (39.1)	134 (60.9)	
	35–49	192	85 (44.3)	107 (55.7)	
Education					
	No education	73	29 (39.7)	44 (60.3)	0.75
	Primary	312	119 (38.1)	193 (61.9)	
	Secondary & above	104	36 (34.6)	68 (65.4)	
Marital status					
	Married/Cohabiting	351	132 (37.6)	219 (62.4)	1.00
	Others	138	52 (37.7)	86 (62.3)	
Region					
	Northern	67	25 (37.3)	42 (62.7)	0.77
	Central	109	38 (34.9)	71 (65.1)	
	Southern	313	121 (38.7)	192 (61.3)	
Residence					
	Urban	127	45 (35.4)	82 (64.6)	0.59
	Rural	362	139 (38.4)	223 (61.6)	
Currently working					
	Yes	329	56 (35.0)	104 (65.0)	0.42
	No	160	128 (38.9)	201 (61.1)	
Wealth index					
	Poorest	64	27 (42.2)	37 (57.8)	0.44
	Poorer	77	30 (39.0)	47 (61.0)	
	Middle	82	24 (29.3)	58 (70.7)	
	Richer	109	45 (41.3)	64 (58.7)	
	Richest	157	58 (36.9)	99 (63.1)	
Births in past 5 years					
	0	218	84 (38.5)	134 (61.5)	0.89
	1	190 (38.9)	69 (36.3)	121 (63.7)	
	2 & more	81 (16.6)	31 (38.3)	50 (61.7)	

*Chi-Square test

Multivariable analysis was conducted in three phases. The first model included only socio-demographic variables. The variables that were found to be significantly associated with current practice of FP were secondary and above education with adjusted odds ratio and 95 % confidence interval (AOR, 95 % CI) of 2.06(1.08, 3.91), married/cohabiting women with AOR (95 % CI) of 1.51(1.00, 2.26) and women from the central region with AOR (95 % CI) of 0.53(0.28, 0.99) [Table 5].

**Table 5:** Logistic regression analysis of socio-demographic, reproductive and awareness of HIV positive status variables versus current practice of family planning among HIV infected fecund women

Independent variables	Current practice of any FP		Model 1 adjusted odds ratio (95 % CI)	Model 2 adjusted odds ratio (95 % CI)	Model 3 adjusted odds ratio (95 % CI)
	Yes	No			
Education					
No formal education	29	44	Reference	Reference	Reference
Primary	165	147	1.69(0.99,2.87)	1.53(0.87,2.68)	1.61(0.91,2.85)
Secondary & above	57	47	2.06(1.08,3.91)^a^	2.17(1.08,4.36)^a^	2.36(1.16,4.78)^a^
Marital status					
Married/Cohabiting	190	161	1.51(1.00,2.26)^a^	1.17(0.76,1.81)	1.23(0.79,1.91)
Others	61	77	Reference	Reference	Reference
Region					
Northern	67	42	Reference	Reference	Reference
Central	109	50	0.53(0.28,0.99)^a^	0.51(0.26,0.99)^a^	0.50(0.26,0.98)^a^
Southern	313	159	0.63(0.36,1.09)	0.71(0.40,1.27)	0.69(0.38,1.24)
Residence					
Urban	127	60	0.74(0.48,1.14)	0.93(0.58,1.48)	0.92(0.57,1.48)
Rural	362	191	Reference	Reference	Reference
FP information from radio					
Yes	170	148		1.06(0.71,1.60)	1.10(0.72,1.66)
No	81	90		Reference	Reference
Visited health facility past 12 months					
Yes	207	167		1.52(0.96,2.41)	1.35(0.84,2.15)
No	44	71		Reference	Reference
Births in past 5 years					
0	87	131		Reference	Reference
1	113	77		1.82(1.19,2.79)^b^	2.02(1.30,3.13)^b^
2 & more	51	30		2.03(1.15,3.56)^a^	2.20(1.24,3.91)^b^
No of living children					
0	9	37		Reference	Reference
1–2	103	90		2.99(1.29,6.88)^a^	2.26(0.96,5.32)
3–4	85	73		3.16(1.33,7.48)^b^	2.60(1.08,6.28)^a^
5 & more	54	38		4.12(1.64,10.35)^b^	3.03(1.18,7.82)^a^
Knows HIV positive status					
Yes	119	65			2.32(1.54,3.50)^c^
No	132	173			Reference
–2 Log likelihood of the constant			677.55	677.55	677.55
−2 Log likelihood of the model			662.49	628.55	611.54
Likelihood Ratio Chi^2^ (p-value)			15.06 (0.020)	48.99 (0.000)	66.01 (0.000)
Nagelkerke R^2^			0.040	0.127	0.168
Hosmer & Lemeshow Test Chi^2^ (p-value)			6.40 (0.494)	2.02 (0.980)	11.40 (0.180)

^a^P < 0.05; ^b^p < 0.01; ^c^p < 0.001

In the second model, family planning information from the radio, visit to a health facility, births in the past five years and number of living children were included in addition to the socio-demographic variables in model 1. Secondary and above education and being from the central region maintained significant associations (AOR: 2.17(1.08, 4.36) and 0.51(0.26, 0.99)). Women who had one birth and more than one birth in the past five years were more likely to practice FP as compared to the ones without any birth with AORs (95 % CIs) of 1.82(1.19, 2.79) and 2.03(1.15, 3.56) respectively. The number of alive children the woman had was associated with FP practice: the highest association was between women with at least five alive children [AOR(95 % CI): 4.12(1.64, 10.35)] [Table 5].

In the final model, women’s knowledge of HIV positive status was added on top of the variables included in model 2. Women with secondary and above education were more likely to be practicing FP than women without formal education [AOR (95 % CI): 2.36 (1.16, 4.78)]. Being residents of the central region was associated with a 50 % reduction in the use of FP as compared to northern region residents [AOR (95 % CI): 0.50 (0.26, 0.98)]. Having one birth and more than one birth in the past five years were associated with family planning practice [AOR (95 % CI): 2.02(1.30, 3.13] and [2.20(1.24, 3.91)] respectively. Similarly, the presence of 3–4 and more than 4 alive children were associated with family planning practice with AOR (95 % CI): 2.60 (1.08, 6.28) and 3.03 (1.18, 7.82) respectively. The only variable that was added in the final model, women’s knowledge of HIV positive status, was also found to be statistically significantly associated with FP practice [AOR (95 % CI) of 2.32 (1.54, 3.50)]. Among the variables that were significantly associated with FP practice in the final model, knowledge of HIV positive status showed the strongest association (P < 0.001) [Table 5].

## Discussion

The major finding of this analysis is that women who knew their HIV positive status were more likely to use FP options. A previous prospective study in Malawi showed that the use of contraceptives increased significantly from 38 % before receipt of positive HIV results to 52 % 1-week after receipt of positive HIV results (P < 0.001) [19]. However, the prospective study had a component of FP counseling and the increase in contraceptive use was partly attributed to the counseling. The findings in this analysis are without any counseling or other type of intervention except for women’s exposure to health information/services in their natural settings. This analysis gives natural setting evidence that closely reflects the reality on the ground. If more HIV-infected women know their HIV status, we will achieve a higher level of contraceptive utilization among women living with HIV and hence reduce the risk of MTCT.

The prevention of MTCT of HIV usually focuses on ART, obstetric care and infant feeding practices. Such approaches help to address MTCT among pregnant women who are known to be HIV positive and able to access the health service. Some of the pregnancies among women living with HIV are unintended. Addressing unintended pregnancies among women living with HIV through FP is an area that needs attention. This analysis was carried out to produce evidence on the factors associated with FP among women living with HIV.

A comparison between confirmed HIV-infected women who were aware of their HIV-positive status and those who were unaware of their HIV-positive status was made. The two comparison groups were found to have similar background characteristics except that women in the aware category were older. The two groups did not differ in terms of the number of birth in the past five years (Table 4). Most previous studies compared HIV-infected women to their HIV-uninfected counterparts who were likely to have different background characteristics and risk behaviors. Hence, the current assessment minimizes the significant variations that may exist in the background characteristics of the two groups in comparison and controls the confounding effect of such variability.

The counselling provided to the women who knew their HIV-positive status might have included contraceptive counselling which most likely led to improvement in the knowledge and practice of FP [19]. Despite the expansion in PMTCT services in Malawi, a recent report revealed that a significant proportion of eligible women and babies born to them were not receiving the appropriate medical services [4]. This means that the risk of MTCT exists among the HIV-infected women who are not effectively benefiting from the PMTCT services. Furthermore, studies have shown that a significant proportion of pregnant and post-partum women on ARV do not adhere to their treatment [22]. With poor adherence to ARV, the likelihood of increased MTCT cannot be ruled out. Strengthening FP provision to women living with HIV needs to be given emphasis as it is one of the major strategies in the Malawi EMTCT plan [17]. One of the approaches would be to intensify HIV testing so that more HIV-infected women know their HIV-positive status which may result into an increase in FP practice as it was demonstrated in this study.

The most commonly cited source of information on FP was the radio while TV and newspapers were mentioned by the minority of the respondents. Malawi DHS revealed that 53 % of the households owned radio whereas only 11 % owned television [3]. Radio is widely available in the urban as well as rural settings. These results can be used to guide health managers to target radio over other alternatives as a means to reach the majority of the women with FP messages. Health workers’ home visit was not a significant practice in Malawi. Health care delivery sector needs to strengthen community level health services to improve HIV testing as well as FP uptake. The involvement of health surveillance assistants, community-based distributing agents, village health committees and volunteers would be helpful in accordance with the health sector strategic plan of Malawi [23]. Over three quarters of the women had visited a health facility in the year preceding the study. It is likely that some of the women were already enrolled in HIV care and treatment services that made them visit the health facilities more frequently. Even the women who were unaware of their HIV-positive status might have visited the health facilities for various services related to HIV and opportunistic infections. However, it appeared that there were women who were frequently visiting the health facilities without knowing their HIV positive status. Health facilities need to be vigilant and catch all women by minimizing missed opportunities in HIV testing. Women accessing reproductive health services might have missed the opportunity of receiving HIV-related services like HIV testing and the vice versa. Linkage of reproductive health and HIV programs is of significant importance to coordinate program activities and minimize missed opportunities [24].

The reproductive characteristics were not much different from the general women population in Malawi DHS [3]. Irrespective of their HIV-positive status, couples wished to fulfil their desired family size. Women living with HIV have the right to exercise their reproductive rights, need to have access to services to help them make informed reproductive health decisions and use the available contraceptive options provided they desire to do so [25]. By enabling women living with HIV to prevent or delay pregnancies, and access FP services we can reduce MTCT. In this analysis, one-third of the women were aware of their HIV positive status. The analysis was done on data on HIV-positive women who fulfilled the criteria of recent sexual activity and being non-pregnant. Hence, the proportion of women knowledgeable about their HIV-positive status might not be generalizable to all HIV-infected women as this analysis was made on a sub-population of HIV- infected women.

Knowledge about modern contraceptives was universal and the large majority also reported to have ever used one form of FP method in the past. Current use of FP method was reported by more than half of the women where modern methods were used by the vast majority. In conformity with other studies [26–28], injectable, condom and female sterilization were the mostly used modern methods. Among the users of FP, the most important reason mentioned by the women was limiting the number of children. These results demonstrate that over one-fifth of the women had unmet needs for FP: 7.4 % of unmet need for spacing children and 14.5 % of unmet need to limit. The unmet needs for spacing and limiting the number of children among all women in the reproductive age in the Malawi DHS 2010 report were 14.2 % and 11.9 % respectively [3]. If the unmet need group had accessed FP, a huge number of unwanted pregnancies and possible MTCT could have been averted. Unmet needs for FP is now becoming one of the indicators to assess the quality of PMTCT programs. However, the unmet need in this analysis describes the situation at the population level and not at PMTCT program level.

Better educational background was one of the factors associated with FP use. The third Millennium Development Goal (MDG) (women empowerment), emphasizes on women education which is key to societal development one aspect of which being health as was shown in this case. In Malawi DHS 2010, the highest level of current use of any FP method among women (48 %) was reported in the central region [3]. In contrast, the significant reduction in the use of FP in the central region in this analysis is difficult to explain. The more births in the past five years and the more the number of alive children the woman had, the higher the chance of using FP methods was. Studies done elsewhere among women living with HIV also demonstrated an association between higher number of children and contraceptive use [12, 26–28]. Once the desired fertility level is achieved, women tended to use contraceptives to either space or limit the number of children.

The findings need to be interpreted with the following limitations in mind. The study findings reflect the situation in 2010 and the current status might be different due to the significant scale up of PMTCT program in subsequent years in Malawi. Because this was a secondary data analysis, some important variables were either absent or incomplete like health condition of the women, biological markers of immune status, discussion between couples, stigma and disclosure of HIV status.

## Conclusions

Women’s knowledge of their HIV positive status was found to be a significant predictor of their FP practice. Health managers and clinicians need to improve HIV counselling and testing coverage among women of child- bearing age and address the FP needs of HIV-infected women. HIV/AIDS and Reproductive Health programs should be integrated and complement each other.
